# Hyperspectral infrared nanoimaging of organic samples based on Fourier transform infrared nanospectroscopy

**DOI:** 10.1038/ncomms14402

**Published:** 2017-02-15

**Authors:** Iban Amenabar, Simon Poly, Monika Goikoetxea, Wiwat Nuansing, Peter Lasch, Rainer Hillenbrand

**Affiliations:** 1 CIC nanoGUNE, 20018 Donostia-San Sebastián, Spain; 2 IFIB - Interfaculty Institute of Biochemistry, 72076 Tübingen, Germany; 3 Metallic Surfaces Unit, IK4-CIDETEC, 20014 Donostia-San Sebastián, Spain; 4 Department of Proteomics and Spectroscopy (ZBS6), Robert Koch Institut, 13353 Berlin, Germany; 5 CIC NanoGUNE and UPV/EHU, 20018 Donostia-San Sebastián, Spain; 6 IKERBASQUE, Basque Foundation for Science, 48013 Bilbao, Spain

## Abstract

Infrared nanospectroscopy enables novel possibilities for chemical and structural analysis of nanocomposites, biomaterials or optoelectronic devices. Here we introduce hyperspectral infrared nanoimaging based on Fourier transform infrared nanospectroscopy with a tunable bandwidth-limited laser continuum. We describe the technical implementations and present hyperspectral infrared near-field images of about 5,000 pixel, each one covering the spectral range from 1,000 to 1,900 cm^−1^. To verify the technique and to demonstrate its application potential, we imaged a three-component polymer blend and a melanin granule in a human hair cross-section, and demonstrate that multivariate data analysis can be applied for extracting spatially resolved chemical information. Particularly, we demonstrate that distribution and chemical interaction between the polymer components can be mapped with a spatial resolution of about 30 nm. We foresee wide application potential of hyperspectral infrared nanoimaging for valuable chemical materials characterization and quality control in various fields ranging from materials sciences to biomedicine.

## Abstract (web summary)

In hyperspectral imaging a broadband spectrum is recorded at each pixel, which creates information-rich images. Here, the authors combine this concept with Fourier transform infrared nanospectroscopy to achieve 5,000-pixel, nanoscale-resolution images at wavelengths between 5 and 10 micrometres.

Infrared (IR) vibrational spectroscopy^1^ is a valuable tool for materials characterization in widely different fields, ranging from polymer sciences to biomedical imaging^2^,^3^,^4^,^5^. It allows for highly sensitive studies of chemical and structural properties, among others. Diffraction, however, limits its spatial resolution and sensitivity, thus preventing the study of nanoscale materials and composites, as well as of single biological macromolecules. The IR diffraction limit can be circumvented, among other techniques^6^,^7^,^8^ by infrared scattering-type scanning near-field optical microscopy (IR s-SNOM)^9^,^10^ and its extension to nanoscale Fourier transform infrared (nano-FTIR) spectroscopy^11^,^12^,^13^,^14^,^15^,^16^,^17^,^18^,^19^, which enable infrared imaging and spectroscopy with nanoscale spatial resolution, respectively.

s-SNOM and nano-FTIR spectroscopy are based on atomic force microscopy (AFM), where either monochromatic or broadband infrared radiation, respectively, is scattered by a metallic AFM tip. The tip acts as an antenna and concentrates the incident infrared field at the very tip apex to a nanoscale spot size (nanofocus)^10^,^12^,^20^, locally exciting infrared-vibrational resonances in the sample that modify the tip's scattered field. In s-SNOM, the tip-scattered field is recorded with an interferometer as a function of tip position, yielding two-dimensional (2D) monochromatic infrared amplitude and phase images. The spatial resolution is determined by the radius of the tip apex, which is typically in the range of 10–30 nm. In nano-FTIR spectroscopy, the tip is illuminated with the radiation from a thermal source^12^,^20^,^21^, an infrared laser continuum^11^,^14^,^22^,^23^,^24^,^25^ or a synchrotron^17^,^18^,^19^,^26^. Fourier transform spectroscopy of the scattered light yields a local infrared-vibrational spectrum (point spectrum) of the sample surface with a spatial resolution similar to s-SNOM. Because of an asymmetric spectrometer design (that is, tip and sample are located in one of the interferometer arms^14^,^16^,^23^), both amplitude and phase (respectively real and imaginary part) of the scattered field are measured. It has been shown that the phase, respectively imaginary part of nano-FTIR spectra of organic materials can be well interpreted with the help of far-field FTIR absorbance spectra, allowing for materials characterization and identification based on standard infrared references^14^,^15^,^18^,^27^.

Spatially resolved spectroscopic information can be obtained by sequential s-SNOM imaging of one and the same sample area at different infrared frequencies using a tunable monochromatic laser. From the stack of images, an infrared spectrum can be constructed at each pixel^28^,^29^,^30^,^31^,^32^,^33^,^34^. Typically, tunable monochromatic CO_2_ or cw-quantum cascade lasers are used as infrared source. As they can be tuned only over a relatively small spectral width of about 200 cm^−1^, several lasers are required to cover the whole mid-infrared spectral range. On the other hand, frequencies below 900 cm^−1^ are barely reached with table-top cw-lasers. Further, the nonlinear and typically uncontrolled drift between sample scanner and tip, as well as the tip wear due to the numerously repeated sample scanning, is challenging the construction of accurate local broadband infrared spectra.

Spectroscopic infrared nanoimaging, in principle, can be also achieved by recording a nano-FTIR spectrum at each pixel of a 2D image. Advantages would include the availability of continuous broadband radiation also below 900 cm^−1^ (provided by thermal sources^12^,^21^, synchrotrons^18^,^26^,^35^ and infrared laser continua^14^,^15^,^22^,^23^,^36^,^37^), and that repetitive scanning and related problems (tip wear) could be avoided. However, the low-spectral irradiance of many broadband infrared sources is strongly challenging nano-FTIR based hyperspectral infrared nanoimaging, that is, the recording of a large 2D array of nano-FTIR spectra. For example, synchrotrons provide ultra-broadband infrared radiation with a spectral irradiance of about 0.2 W cm^−2^ cm^−1^ (ref. 18) at mid-infrared frequencies, which is several orders of magnitude weaker than that of mid-IR quantum cascade lasers used for s-SNOM imaging. The reported time for recording nano-FTIR spectra of materials with a strong infrared response (for example, phonons in SiO_2_) amounts to about 1 min (ref. 18), which is significantly increased for weakly absorbing organic substances. The recoding of a hyperspectral image of about 100 × 100=10,000 pixel would require many tens of hours, thus challenging the mechanical and optical stability of nano-FTIR setups. Alternatively to synchrotrons, mid-infrared laser continua based on difference frequency generation (DFG) can be applied for nano-FTIR spectroscopy^14^,^15^,^22^,^23^,^36^,^37^. These table-top lasers provide tunable infrared radiation of several 100 cm^−1^ bandwidth and a spectral irradiance that can be much higher compared with synchrotrons. However, hyperspectral infrared nanoimaging with such lasers has not been achieved yet. Reasons include the non-negligible sample drift during spectra acquisition, as well as the still relatively small spectral bandwidth that requires the combining of multiple bandwidth-limited nano-FTIR spectra at each pixel.

Here we introduce and describe hyperspectral infrared nanoimaging by nano-FTIR spectroscopy with a tunable laser continuum. It is based on recording and stitching together multiple bandwidth-limited nano-FTIR spectra at each pixel of a 2D sample area, which is enabled by sample drift correction during data acquisition. Specifically, we use a tunable DFG laser continuum (350 cm^−1^ effective bandwidth) to record nanoscale-resolved hyperspectral infrared images comprising about 5,000 nano-FTIR spectra that cover the spectral range from 1,000 to 1,900 cm^−1^. We verify the technique with a three-component polymer blend and a hair cross-section, and demonstrate that standard multivariate analysis of the hyperspectral images can be performed. As a first application, we demonstrate the much sought-after possibility of mapping chemical interaction between polymer components with nanoscale spatial resolution.

## Results

### Set-up and methodology for hyperspectral infrared nanoimaging

**Figure 1:**
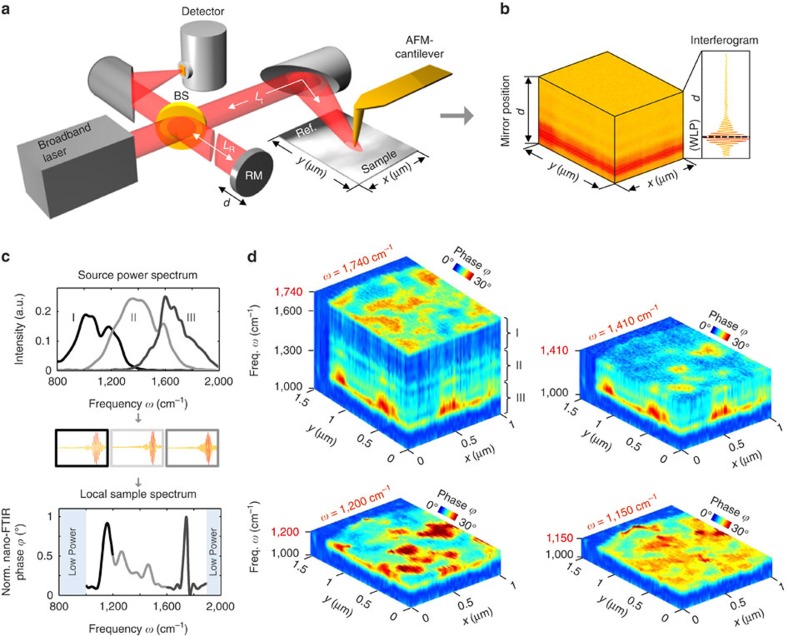
Hyperspectral infrared nanoimaging. (**a**) Set-up employing a mid-infrared laser continuum source for tip illumination. The tip scans the sample surface while at each pixel the light backscattered from the tip is analysed with a Michelson interferometer that is operated as a Fourier transform spectrometer. The setup comprises a beam splitter (BS, uncoated ZnSe), a reference mirror (RM) and a detector. (**b**) 2D array of interferograms. (**c**) Top: Output spectra of the source. Middle: Interferograms recorded for each output spectrum. Bottom: Broadband nano-FTIR spectrum composed by three nano-FTIR spectra obtained by Fourier transform of the corresponding interferograms and normalization to a reference spectrum (middle panel). (**d**) Hyperspectral infrared data cubes of spectral resolution of 35 cm^−1^, cut at different frequencies *ω*. They show the normalized phase *ϕ* of the tip-scattered light as a function of position (*x*, *y*) and frequency *ω*.

We developed hyperspectral infrared nanoimaging (Fig. 1) using a commercial nano-FTIR set-up (Neaspec GmbH). It is based on an AFM, where a standard Au-coated tip is vertically vibrating at the mechanical resonance frequency Ω of the cantilever. The tip is illuminated with a DFG-generated mid-infrared laser continuum of about 350 cm^−1^ spectral bandwidth, which centre frequency is tuneable between 1,200 and 1,600 cm^−1^ (see Fig. 1a and Supplementary Note 1). The tip-scattered light is recorded with a Michelson interferometer. To perform background-free nano-FTIR spectroscopy^14^,^38^,^39^, the detector signal is demodulated at a higher harmonic *n* of the tip's oscillation frequency, *n*Ω, and recorded as a function of the reference mirror position *d*, yielding the interferogram *I*(*d*). Throughout this work, the demodulation order was *n*=3. Because tip and sample are located in one interferometer arm, Fourier transform of *I*(*d*) yields amplitude *s*_s_(*ω*) and phase *ϕ*_s_(*ω*) spectra^14^,^27^. To obtain normalized nano-FTIR spectra *s*=*s*_s_/*s*_ref_ and *ϕ=ϕ*_s_*−ϕ*_ref_, the tip is positioned on a reference area on the sample (typically a clean gold or silicon surface)^14^,^23^ to record the reference spectra *s*_ref_(*ω*) and *ϕ*_ref_(*ω*).

For hyperspectral nanoimaging we record interferograms at each pixel (*x*, *y*) of a 2D area of the sample surface (Fig. 1b). Subsequent Fourier transform and normalization to reference spectra yields a 2D array of nano-FTIR spectra with a spectral bandwidth determined by the output spectrum of the DFG laser source, that is, a hyperspectral data cube *A*(*x*, *y*, *ω*) where *x* and *y* represent two spatial dimensions and *ω* the frequency (spectral dimension). For the present work, we recorded and studied nano-FTIR phase spectra *ϕ*(*ω*), as they are related to the sample's infrared absorption^16^,^27^, the later being typically analysed when infrared spectroscopy of organic materials is performed. To increase the spectral bandwidth, we record data cubes *A*^k^=*ϕ*^k^(*x*, *y*, *ω*) at three different DFG output spectra (indicated by the index k=I,II,III and shown in the upper panel of Fig. 1c) and stitch together at each pixel the corresponding normalized nano-FTIR phase spectra (Fig. 1c, lower panel). As a result, a hyperspectral data cube *A* is obtained. In Fig. 1d we show the hyperspectral infrared data cube of a three-component polymer blend on a silicon substrate, exhibiting a spatial resolution of about 30 nm (further details see below). Cutting the cube at different infrared frequencies *ω* yields monochromatic infrared images. They clearly reveal a rich variety of spectrally and spatially varying features, indicating that neither individual point spectra nor individual monochromatic images provide the full information content contained in the hyperspectral data of this sample.

In the following we describe the key implementations of our technique (for more technical details see Supplementary Notes 1–6).

We increased the data acquisition speed by improving the signal-to-noise ratio (SNR) of the individual nano-FTIR spectra. To that end, the output power of our mid-IR laser continuum (previously reported in refs 14, 23) was increased from about 100–250 μW to 600 μW. Further, because of the asymmetric interferometer set-up (that is, the sample is located in one of the interferometer arms), we record only one half of the interferogram (Fig. 1b), as the other one does not contain spectroscopic information about the sample (see Supplementary Note 2 and Supplementary Fig. 1). With our improved laser source we succeeded to obtain about 350 cm^−1^ broad nano-FTIR spectra of organic materials in 1.66 s, which is more than one order of magnitude faster than what has been previously achieved with DFG and synchrotron radiation^14^,^18^,^23^.

For accurate and reliable normalization of the nano-FTIR spectra, the reference spectra need to be recorded under the same experimental conditions. Due to slight spectral fluctuations of the laser continuum and drift of the interferometer arms (see discussion below, Supplementary Note 5 and Supplementary Fig. 3), however, the conditions may vary between sample and reference measurements. For that reason, we regularly acquire interferograms of a reference area while recording the data cube. This can be achieved, for example, by recording the data cube such that each line contains a clean reference area (for example, silicon, marked Ref. in Fig. 1a where the line scans are parallel to the *y*-axis). The nano-FTIR spectra of each line are then normalized to the reference spectrum included in this line.

Depending on the number of individual spectra, the acquisition of a data cube *A*^k^ may still take several minutes to few hours. To avoid artifacts due to sample drift, we adapted concepts known from other imaging techniques (for example, electron energy loss spectroscopy mapping or AFM^40^). In brief, after *n* spectroscopic line scans (that is, recoding nano-FTIR spectra along *n* complete lines of the image), the sample is repositioned, yielding a data cube in which sample drift has been compensated (see Supplementary Note 3 and Supplementary Fig. 2). To this end, the spectroscopic data acquisition is stopped after each block of *m* line scans and a topography image of the sample including a reference point is recorded. The position of the reference point relative to its previous position is measured. The sample scanner is accordingly repositioned. We chose the number *m* such that the sample drift (determined essentially by the temperature stability of the setup) during the *m* line scans is smaller than the spatial resolution of about 30 nm. In the experiments presented in Figs 3 and 4 we set *m*=2 and *m*=4, respectively.

To combine the individual bandwidth-limited data cubes *A*^k^, we need to combine at each position (*x*, *y*) the individual phase spectra *ϕ*^k^(*x*, *y*, *ω*) obtained with the different DFG settings. A key for achieving this task is the sample drift correction during the recording of each bandwidth-limited data cube. As outlined above, the sample drift correction ensures that the position uncertainty is less than the spatial resolution, thus ensuring that spectra of the same position (*x*, *y*) are combined.

**Figure 2:**
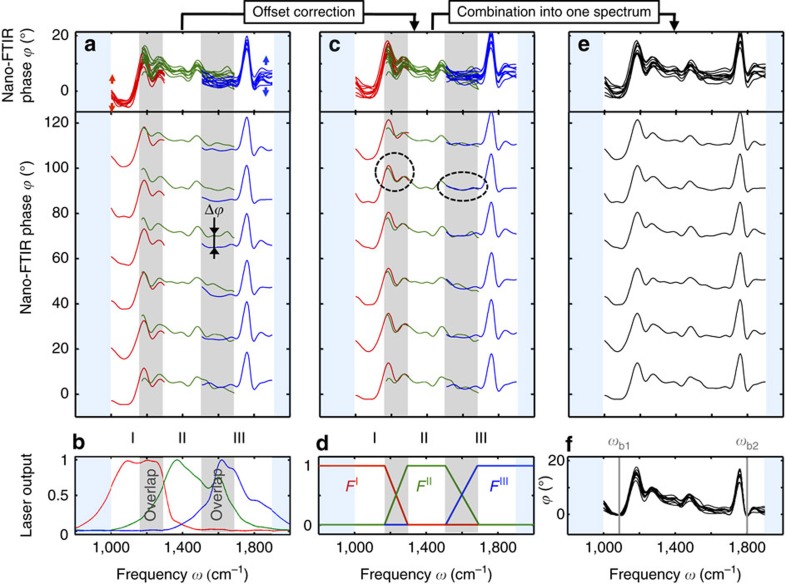
Stitching of bandwidth-limited normalized nano-FTIR spectra. (**a**) Bandwidth-limited normalized nano-FTIR phase spectra from six subsequent pixels of the hyperspectral data cube shown in Fig. 1. (**b**) Laser output spectra. (**c**) Spectra of **a** after offset correction. (**d**) Functions *F*^I^, *F*^II^ and *F*^III^. (**e**) Broadband nano-FTIR spectra after stitching. (**f**) Broadband nano-FTIR spectra after stitching and baseline correction. The top panels of **a**,**c**,**e** show all normalized spectra plotted on top of each other, while the bottom panels show the same spectra plotted for each pixel separately.

The remaining challenges and solutions for combining the individual spectra are shown in Fig. 2. We recorded sample-drift-corrected data cubes of the same sample area using the three different laser outputs shown in Fig. 2b (numerated k=I, II, III). In Fig. 2a (bottom) we show the bandwidth-limited nano-FTIR phase spectra *ϕ*^k^(*x*, *y*, *ω*) of six subsequent pixels on the polymer blend sample. For better comparison, and to demonstrate the reproducibility of the individual spectra, the upper panel of Fig. 2a displays all spectra plotted on top of each other. Within the overlapping spectral regions (marked by grey areas) we clearly observe the same spectral features in the adjacent bandwidth-limited nano-FTIR phase spectra. However, the spectra can be significantly offset against each other by up to 12 degrees, although all spectra are normalized to a reference spectrum (see above). We explain this phase offset Δ*ϕ* (marked in Fig. 2a) by a small unavoidable drift of the interferometer paths (*L*_R_ and *L*_t_ in Fig. 1a), which occurs between the acquisition of the individual nano-FTIR spectra and the acquisition of the reference spectrum (see Supplementary Note 5 and Supplementary Fig. 3). A drift as small as 100 nm of *L*_R_ relative to *L*_t_ shifts the normalized nano-FTIR phase spectrum by about Δ*ϕ*=6 degree, which is in the same order of magnitude as the phase shift produced by absorption in the sample. To reduce offset fluctuations below 1 degree, the path lengths *L*_R_ and *L*_t_ (of about 6 cm) need to be stabilized with a precision better than 20 nm, which, however, will require sophisticated technology development in the future. Here we tackled the problem by correcting the phase offset. To that end, we shift at each pixel (*x*, *y*) the phase spectra *ϕ*^I^(*ω*) and *ϕ*^*III*^(*ω*) by the constant phase values 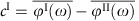 and 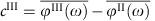 (the average values are evaluated in the corresponding spectral overlap regions marked grey in Fig. 2), to match *ϕ*^II^(*ω*). The offset-corrected phase spectra are shown in Fig. 2c. We find that the spectral features (marked by dashed black circles) in the overlapping regions are now well matched for all pixels, thus verifying the validity and reliability of our rather simple offset-correction procedure. Finally, we combine the three offset-corrected spectra at each pixel to obtain a single-broadband nano-FTIR phase spectrum. To achieve a smooth transition between the spectral ranges, we multiply the phase spectra *ϕ*^k^ by the functions *F*^k^(*ω*) shown by the red, green and blue graphs in Fig. 2d. Subsequently, the spectra are summed up. The final broadband nano-FTIR phase spectra are shown in Fig. 2e. Altogether, this methodology enables an automatized combination of bandwidth-limited data cubes to one hyperspectral data cube.

Analogue to far-field FTIR spectroscopic imaging, we finally apply a baseline correction^41^,^42^ to each broadband (composite) nano-FTIR phase spectrum *ϕ*(*ω*). We select two frequencies *ω*_b1_ and *ω*_b2_ (marked in Fig. 2f, where we know or can assume that the sample absorption is negligible, see Supplementary Fig. 5) and subtract the linear baseline defined by the phase values *ϕ*(*ω*_b1_) and *ϕ*(*ω*_b2_). A comparison of baseline-corrected spectra (Fig. 2f) with the corresponding uncorrected spectra (Fig. 2e, upper panel) clearly shows the reduction of fluctuations between neighbouring spectra. Hence, the baseline correction significantly improves the SNR in the images extracted from the hyperspectral data cube (see Supplementary Note 6 and Supplementary Fig. 4).

The recording of a bandwidth-limited data cube *A*^k^ consisting of 5,084 nano-FTIR spectra (that is, 82 × 62 pixels, Fig. 1d) took (5,084 × 1.66 s)/3,600=2.3 h while the accumulated additional time needed for the sample repositioning was of less than 10 min (for more details see Supplementary Note 3). Thus, the recording of the hyperspectral data cube *A* shown in Fig. 1d required 7.4 h.

### Hyperspectral chemical nanoimaging of a polymer blend

**Figure 3:**
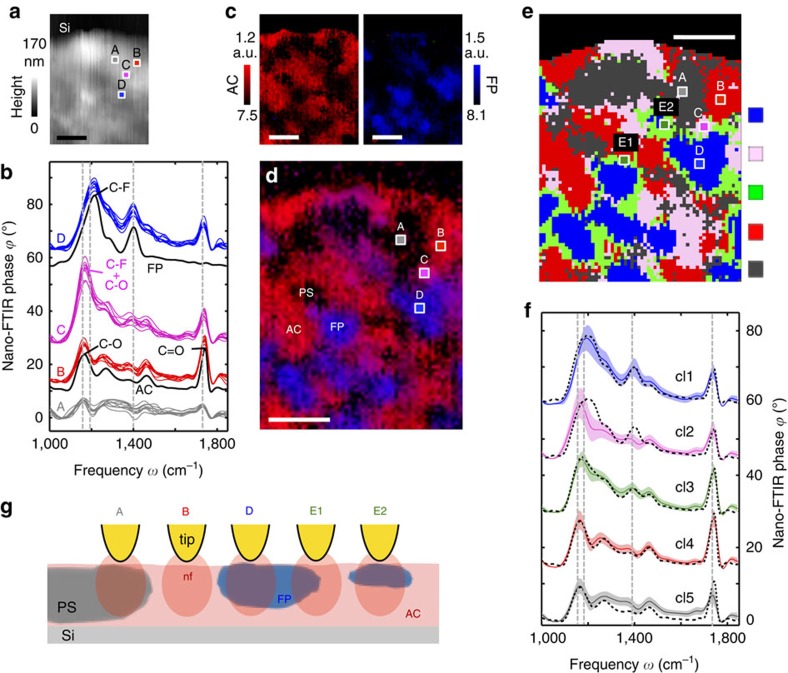
Chemical nanomapping of polymers. (**a**) Sample topography. Scale bar, 400 nm. (**b**) Black curves show nano-FTIR spectra of FP and AC reference samples (44 min total acquisition time; 35 cm^−1^ resolution). Coloured curves show the nine nano-FTIR spectra in the areas marked A, B, C and D in **a** and **d**, extracted from the data cube shown in Fig. 1d. All spectra are vertically offset. (**c**) Spatial maps of AC and FP obtained by inter-spectral distance mapping. Scale bar, 400 nm. (**d**) Compositional map, obtained by superposition of the maps of **c**. Scale bar, 400 nm. (**e**) Cluster map obtained by multivariate data analysis (hierarchical cluster analysis, HCA) showing clusters from cl1 to cl5 (blue, pink, green, red and dark grey, respectively). Scale bar, 400 nm. (**f**) Average nano-FTIR spectra and s.d. of clusters cl_i_ (coloured thin and thick lines, respectively). Black dashed spectra show best fits obtained by linear superposition of the pure FP and AC reference nano-FTIR spectra shown in **b**. (**g**) Schematics of near-field probing at positions A-E2 marked in **d** and **e**.

In a first application example, we demonstrate hyperspectral IR nanoimaging with a three-component polymer blend, showing that this novel tool can meet the strong demand for highly sensitive nanoscale chemical mapping of the spatial distribution and local chemical interaction of the components. The model system studied in this work is based on a fluorine copolymer (FP), an acrylic copolymer (AC), and a polystyrene latex (PS; for details and fabrication see ‘Methods' section). Figure 3a shows the topography image of the about 170 nm thick spin-coated polymer blend on silicon, while the hyperspectral infrared data of this sample area are displayed in Fig. 1d. The good reproducibility of the individual spectra (see Fig. 3b showing four sets of neighbouring point spectra at the sample positions A to D marked in Fig. 3a,d) allow for valuable multivariate analysis based on established procedures known from far-field IR spectroscopy, as we demonstrate in the following.

We first applied inter-spectral distance mapping, where we calculate for each pixel spectrum S(*x*, *y*) the multivariate spectral distance D(*x*, *y*) between the point spectrum and a reference spectrum (see ‘Methods' section). The distance values are converted into colour scales and plotted as specifically coloured pixels at the positions (*x*, *y*). In the resulting distance maps, high colour intensity denotes a small distance (high similarity) with the reference spectrum (and vice versa). With nano-FTIR spectra obtained from reference samples made of pure AC and FP components (black spectra in Fig. 3b) we obtained the distance (similarity) maps for the AC (red) and FP (blue) components shown in Fig. 3c. Superposition of the two maps (see ‘Methods' section) yields a compositional map (Fig. 3d), which highlights areas with the highest relative content of each polymer. We observe homogenous but distinct red and blue areas for the two different references, indicating that the AC and FP components are not fully mixed but rather separated. On the red areas, representative spectra (B) match well with the AC reference (black reference spectrum in Fig. 3b), indicating the presence of pure AC (illustrated by situation B in Fig. 3g). Within the blue areas, interestingly, representative spectra (D in Fig. 3b) show FP peaks at low frequencies (*ω*<1,500 cm^−1^) but also the C=O peak of AC at 1,740 cm^−1^. It can be shown (Supplementary Fig. 6) that the spectra at position D are a linear superposition of the pure FP and AC spectra, which lets us conclude that both FP and AC are present within the volume probed by the near field below the tip apex (represented in Fig. 3g by the reddish elliptical area below the tip apex). We conclude that FP forms cluster-like nanostructures, while AC is spread all over the sample surface. For that reason, the near field below the tip apex probes both the FP cluster and AC layer below the FP cluster (illustrated by situation D in Fig. 3g). We explain this finding by the differences in viscosity, molecular weight and/or chain stiffness. On the other hand, the clearly visible spatial dispersion of AC and FP—avoiding the formation of percolation networks—indicates that AC and FP are miscible even at the nanoscale. Note that any improvement of film formation or film homogenization by mixing or optimizing drying conditions was beyond the scope of this study.

In Fig. 3d we also observe black areas on the polymer blend, indicating that the similarity of the local spectra (A in Fig. 3b) is low compared with the FP and AC reference spectra. We explain these areas by the dominating presence of PS (illustrated by situation A in Fig. 3g). Note that we did not perform distance mapping with PS references, as the peaks in nano-FTIR spectra of pure PS reference sample are comparably weak.

Interestingly, Fig. 3d reveals purple areas (marked C in Fig. 3d), which indicate local spectral differences compared with both the AC (red) and FP (blue) regions. Indeed, representative spectra (marked C in Fig. 3b) of the purple areas cannot be reproduced by a linear superposition of AC and FP reference spectra (see Supplementary Fig. 6). Compared with the red AC spectrum (B), the C=O peak at 1,740 cm^−1^ is reduced, indicating that the amount of AC in the probing volume of the tip is reduced. On the other hand, the peak at 1,155 cm^−1^ is significantly increased. We attribute this finding to the presence of FP and its chemical interaction with AC. Note that chemical interactions are known to cause peak shifts^33^ and increase of peak heights^43^. Specifically, we assume a spectral shift of the CF_2_ stretching vibration of FP from 1,195 cm^−1^ towards lower frequencies, most likely due to the formation of hydrogen bonding between the C-F bonds of FP and the acrylic polymer chains^44^,^45^. Further, the C-O stretching of the ester bond of AC at 1,155 cm^−1^ may be enhanced due to the chemical interaction. The concerted action of both effects could thus explain the enhanced peak at 1,155 cm^−1^ that is found in the purple regions, which consequently indicate the areas where the AC and FP components are well mixed.

We note that similar compositional maps as the ones of Fig. 3c,d can be obtained with far-field infrared reference spectra of the pure AC and FP samples, as we demonstrate in the Supplementary Fig. 7 with the help of attenuated total reflectance FTIR (ATR-FTIR) spectra. This possibility enables rapid hyperspectral data analysis (that is, identification of specific target components) based on standard references without the need of nano-FTIR reference spectra.

Next we demonstrate that valuable multivariate data analysis can be also performed without any reference spectra. To this end, we apply unsupervised hierarchical cluster analysis^46^ (see ‘Methods' section), which was used to segment the hyperspectral data of Fig. 1d into five distinct clusters (denoted as cl1 to cl5, see Fig. 3e). We justify the choice of at least four clusters by our findings from inter-spectral distance mapping (Fig. 3d). On the other hand, segmentation into more than five clusters resulted essentially in further segmentation of the PS-rich (dark) areas. In the future, a more detailed analysis could be applied to determine the optimal number of clusters^47^, which, however, would go beyond the scope of this work. The cluster map is shown in Fig. 3e, and the cluster spectra (coloured) in Fig. 3f. The spectra and areas of clusters cl1, cl2, cl4 and cl5 agree well with Fig. 3b,d, respectively, corroborating the robustness and reliability of the data and the strategy of data analysis. Interestingly, unsupervised cluster analysis reveals another significant area (cluster cl3, green) which is not recognized in Fig. 3d, and which average spectrum (green curve in Fig. 3f) can be reconstructed by a linear superposition of AC and FP reference spectra. We explain it by either a lateral (illustrated by situation E1 in Fig. 3g) or vertical (illustrated by situation E2 in Fig. 3g) arrangement of AC and FP within the volume probed by the tip's near field. For that reason, the green areas indicate interfacial areas without chemical interaction, in contrast to the purple regions where peak shifts indicate significant chemical interaction. Note that polymer chain interactions depend on several factors such as distance between interacting groups, orientation or steric hindrance^43^,^44^, and thus may occur only partially at the interface between AC and FP.

### *In situ* analysis of native melanin in human hair medulla

**Figure 4:**
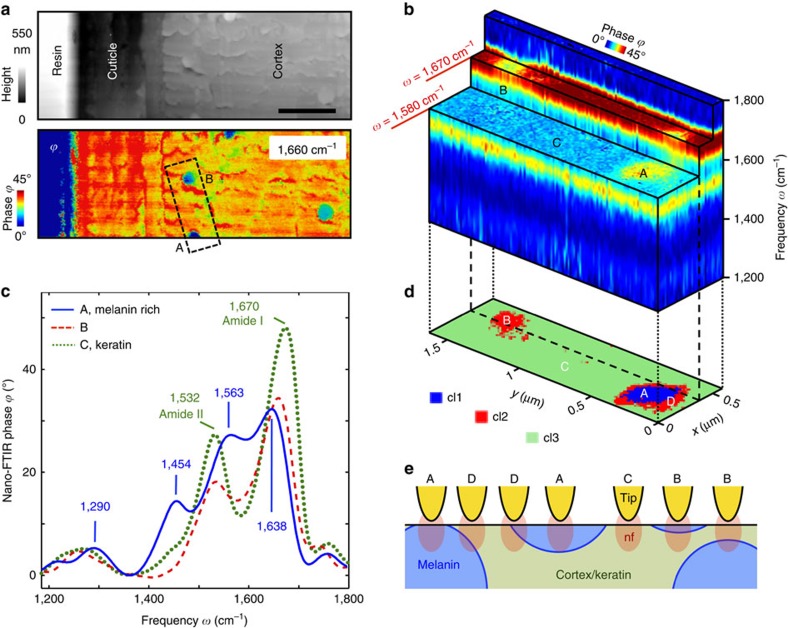
*In situ* hyperspectral infrared nanoimaging of native melanin in human hair. (**a**) Top: Topography of a resin-embedded human hair microtome cross-section. Bottom: Infrared near-field phase image *ϕ* at 1,660 cm^−1^. Scale bar, 1 μm. (**b**) Hyperspectral infrared data cube with a spectral resolution of 35 cm^−1^ partially cut at different frequencies *ω*. It shows the phase of the tip-scattered light as a function of position (*x*, *y*) and frequency *ω*. The dashed black rectangle in **a** shows the area where the data cube was recorded. (**c**) Nano-FTIR spectra (average over nine neighbouring pixels) at positions marked by A, B and C in **a**,**b** and **d**, extracted from the data cube shown in **b**. (**d**) Cluster map obtained by multivariate data analysis (HCA algorithm). (**e**) Schematics of near-field probing at positions A–D marked in **d**.

In Fig. 4 we apply hyperspectral IR nanoimaging to perform the first *in situ* infrared-vibrational chemical analysis of native melanin in human hair medulla. Melanin is a polymer pigment, present in the human hair and skin, and responsible for tissue colouring and ultraviolet photoprotection^48^. Due to its photo absorbing properties, melanin has attracted large attention from cosmetic and solar energy industries^49^,^50^. Unfortunately, it has revealed impossible to analyse human melanin without extracting it from hair or tissue, which comes along with potential damage and modification^51^,^52^.

Figure 4a shows the topography of a resin-embedded cross-section of a hair. In the infrared near-field image taken with a quantum cascade laser at 1,660 cm^−1^ we observe an enhanced infrared absorption of the cuticle and cortex regions compared with the resin, owing to the strong amide I absorption of the hair proteins (α-keratin microfibrils). Within the cortex region we find disk-shaped areas of about 300 nm diameter, where the infrared absorption is reduced (that is, the protein content is reduced). Their size and distribution corresponds to that of melanin granules observed in electron microscopy images^53^. However, we found that nano-FTIR spectra of the individual granules can differ significantly from each other. To elucidate the spectroscopic variations, we performed hyperspectral IR nanoimaging of the area marked by dashed black line in Fig. 4a. From the hyperspectral data cube (Fig. 4b) we extracted spectra at different positions (Fig. 4c). Within the cortex region (position C, green spectrum in Fig. 4c) we observe the well-known amide I and II bands being typical for protein (α-keratin). For particle A (blue curve in Fig. 4c) we observe four distinct peaks that are characteristic for melanin: at 1,290 cm^−1^ (–C–OH phenolic stretching), 1,454 cm^−1^ (C–C aliphatic stretches), 1,563 cm^−1^ (indole N–H bending) and 1,638 cm^−1^ (C=C, C=O, and/or COO- stretching in aromatic cycle)^54^. On particle B—supposed to be a melanin granule—the nano-FTIR spectrum significantly differs from that of particle A. Surprisingly, spectrum B shows more similarity to the keratin spectrum of the cortex region (C), although the amid I and II peaks are shifted by several cm^−1^. Further, while both particles A and B are seen in the monochromatic image at 1,670 cm^−1^, only particle A exhibits a contrast at 1,580 cm^−1^ (see corresponding slices of the data cube in Fig. 4b). Obviously, a set of images and local spectra is not sufficient to clarify the identity of particle B. However, having acquired a full hyperspectral data cube, we can take advantage of multivariate data analysis. Since there are no reference spectra available for natural melanin in hair, we performed unsupervised hierarchical cluster analysis of the hyperspectral data. The segmentation map resulting from cluster analysis with three clusters (Fig. 4d) reveals well-defined features, thus corroborating the applicability and robustness of cluster analysis to the nano-FTIR spectra. We note that cluster analysis with more than three clusters does not reveal well-defined new features (see Supplementary Fig. 8). In both maps the particles A and B appear as homogenous, although as distinct clusters (circular blue and red areas, respectively). Most important, we find a red ring (D) around the blue central area of particle A, revealing that the corresponding spectra belong to the same cluster as those of particle B. It can be shown that the spectra of the red cluster (B and D) are a linear superposition of spectra A and C (see Supplementary Fig. 9). For the width of the red ring (D) we measure about 50 nm, which is in the range of the lateral spatial resolution. We thus conclude that the red ring highlights a steep interface between melanin and keratin (see illustrations D in Fig. 4e). The red area B, in contrast, is a closed disk-shaped area of about 200 nm diameter (that is, larger than the spatial resolution). We thus conclude that spectra B of this area are due to a vertical arrangement of melanin and keratin, that is, a horizontally oriented interface between them. Indeed, near-field probing can be sensitive to subsurface components^55^, which lets us conclude that particle B is either a subsurface melanin granule or a thin slice of a melanin granule (see illustrations B in Fig. 4e; note that melanin granules are elliptical vesicles of an aspect ratio of about 3:1).

## Discussion

The results presented in Figs 3 and 4 clearly demonstrate that hyperspectral data cubes of several 1,000 infrared spectra can be reliably obtained by nano-FTIR spectroscopy using a bandwidth-limited laser continuum. The hyperspectral data allow for multivariate analysis, providing nanoscale maps of the spatial distribution of organic materials. Most important, multivariate analysis reveals two types of spectral peak shifts within the individual nano-FTIR spectra of the polymer and hair samples. In the simplest case, individual nano-FTIR spectra exhibit peak shifts, which can be reproduced by a linear superposition of the nano-FTIR spectra of the pure reference samples (for example, on position D (blue area) on the polymer blend of Fig. 3, or on particle B in the hair sample of Fig. 4). It can be concluded that the spectra originate from the presence of the pure components (identical to that of the reference samples) in the volume probed by the near field below the tip. This can be the case, for example, at a sharp interface between pure components. In the second case, individual nano-FTIR spectra exhibit anomalous peak shifts, which cannot be reproduced by a linear superposition of the spectra of the pure reference samples. We conclude that the spectra originate from the presence of components, which are modified compared with the reference samples. We explain the modified spectral response by a chemical interaction between the adjacent components. Anomalous peak shifts have been found on the purple areas in Fig. 3e, which most likely occurred due to chemical interaction between the FP and AC components of the polymer blend.

Our studies of the hair cross-section of Fig. 4 demonstrate the capacity of hyperspectral infrared nanoimaging to identify and chemically analyse melanin molecules within their granula, without requiring deleterious extraction or tagging processes. Through the analysis of the melanin-specific IR peak position and intensity in the future one could estimate the impact of various cosmetic treatments (like bleaching, ironing or colouring) on the chemical structure of the melanin polymer. Furthermore, analysing the protein secondary structure^23^ could allow for nanoscale spatial mapping of the impact of cosmetic treatments on the keratin fibrils and thus on the hair mechanical resistance.

In summary, we introduced techniques and methods for recording nanoscale-resolved hyperspectral infrared images obtained with a tunable bandwidth-limited mid-infrared laser continuum. It relies on recording multiple bandwidth-limited data cubes of one and the same sample area, and stitching them together to one hyperspectral data cube. Key for the reliable and accurate stitching of the data cubes has been the implementation of methods for (i) regular sample drift correction during data acquisition, (ii) regular recording of reference spectra during data acquisition, (iii) correcting spectral offsets caused by unavoidable interferometer drift and (iv) baseline correction of the broadband nano-FTIR spectra analogous to far-field hyperspectral infrared imaging. Using a tunable DFG laser continuum with an effective spectral bandwidth of about 350 cm^−1^, hyperspectral data cubes of about 5,000 nano-FTIR spectra covering 1,000–1,900 cm^−1^ could be recorded in less than 8 hours. Imaging a three-component polymer blend, we demonstrated that standard multivariate data analysis can be applied to the data, which allows for the automatized generation of chemical maps revealing material clustering and chemical interaction. We also studied a cross section of human hair, demonstrating the possibility of *in situ* multivariate nanoscale infrared analysis of individual melanin granules.

We foresee a wide application potential of hyperspectral infrared nanoimaging in various fields of science and technology, ranging from materials sciences and pharmaceutical applications to biomedical imaging, and from fundamental research to quality control. In combination with multivariate data analysis, hyperspectral infrared nanoimaging yields nanoscale-resolved chemical and compositional maps, including the detection of nanoscale localized chemical interaction. By improving the power and spectral bandwidth of emerging infrared laser sources (a recent work demonstrates a 100 cm^−1^ broad laser continuum of about 100 mW that is tunable between 500 cm^−1^ and 3,000 cm^−1^ ref. 56) and taking advantage of advanced spectral noise reduction strategies (that is, principal component analysis based noise reduction), we envision high-quality, hyperspectral infrared nanoimaging with few wavenumber spectral resolution on a time scale of one hour.

## Methods

### Sample preparation

Three water-based polymer dispersions made by emulsion polymerization were employed in this work. Table 1 summarizes their main characteristics.

Three-component polymer blend for hyperspectral infrared nanoimaging: The three polymer dispersions listed in Table 1 were separately diluted at 0.2% solids content and mixed into a single dispersion by stirring. Then, the mixture was casted onto a silicon wafer through spin coating. Finally, the silicon wafers were dried in an oven overnight at 120 °C. Proportions were chosen in order to obtain a blend for coating applications. The final dispersion contained 40% of a polyvinyledene fluoride (PVDF) and hexafluoropropylene (HFP) copolymer; 40% of an acrylic copolymer made of methyl methacrylate (MMA), butyl acrylate (BA) and acrylic acid (AA); and 20% of a high glass transition temperature (*T*_g_) latex based on polystyrene to improve mechanical properties without the need of any film forming aids^57^.

Polymer reference samples for nano-FTIR spectroscopy: The three polymer dispersions listed in Table 1 were separately diluted at 0.2% solids content. Each one was individually casted onto a silicon wafer through spin coating. Finally, the silicon wafers were dried in an oven overnight at 120 °C.

Polymer reference samples for ATR-FTIR spectroscopy: The three polymer dispersions listed in Table 1 were diluted at 1% and casted onto separate glass substrates and dried overnight at 120 °C. The obtained polymer layers were several μm thick.

### Hair sample preparation

A clean human hair was cut into small pieces and embedded in epoxy resin (Epon kit substitute embedding medium, Aldrich). The sample was cured during 24 h at 60 °C. The block containing the human hair was trimmed with fresh glass knife made with Leica knifemaker and then ultramicrotomed with Leica UCT using Diatome 45° diamond knife.

### FTIR spectroscopy

ATR-FTIR of the hair cross section. We used a Bruker Hyperion 2,000 microscope coupled to a Vertex 70 FTIR spectrometer equipped with an ATR module (× 20 ATR objective, Bruker, single-internal reflection) comprising a germanium crystal with a diameter of about 100 μm at the point of contact with the sample. The spectrum (showing the absorbance) was measured with a spectral resolution of 4 cm^−1^ and presents an average over 250 scans with a total acquisition time of 215 s.

ATR-FTIR of polymers. We used a Frontier FTIR/FIR spectrometer (Perkin Elmer) equipped with a universal ATR module (1 reflection with diamond/ZnSe top plates). The spectra were measured with a spectral resolution of 4 cm^−1^ and present an average over 150 scans with a total acquisition time of 796 s. The reference spectra were recorded on air. All ATR-FTIR spectra show the absorbance.

### Multivariate analysis of hyperspectral infrared nanoimages

Inter-spectral distance mapping (Fig. 3) was carried out by means of the CytoSpec software package (CytoSpec, Berlin, Germany). Distance images were constructed by calculating Euclidean distances between the external reference spectra and the pre-processed nano-FTIR spectra contained in the hyperspectral data sets. In this approach, distance values were converted to colour scales and plotted as a function of the spatial (*x*, *y*) coordinates. In distance imaging, a high colour intensity denotes a low inter-spectral distance, or high similarity, between the actual point spectrum and a given reference spectrum. The CytoSpec software package also allows for constructing so-called composite images that are superpositions of distinct distance maps.

CytoSpec was also used for image reassembling based on cluster analysis (Figs 3 and 4). Basic principles of this image segmentation technique have been published elsewhere^5^,^58^. In this study, *D*-values were used as inter-spectral distance measures and Ward's method was employed as the clustering method. To calculate the multivariate distance values, the spectral range from 1,000 to 1,850 cm^−1^ was utilized.

### Data availability

The data that support the findings of this study are available from the corresponding author upon reasonable request.

## Additional information

**How to cite this article:** Amenabar, I. *et al*. Hyperspectral infrared nanoimaging of organic samples based on Fourier transform infrared nanospectroscopy. *Nat. Commun.* **8**, 14402 doi: 10.1038/ncomms14402 (2017).

**Publisher's note:** Springer Nature remains neutral with regard to jurisdictional claims in published maps and institutional affiliations.

## Supplementary Material

Supplementary Information Supplementary Figures, Supplementary Notes and Supplementary References.

## Figures and Tables

**Table 1:** Main characteristics of the three water-based polymer dispersions employed to prepare the polymer blend.

	Composition (% weight)	Particle diameter, dp (nm)
PVDF/HFP	95/5	129
MMA/BA/AA	49.5/49.5/1	144
PS/AA	99/1	109

AA, acrylic acid; BA, butyl acrylate; HFP, hexafluoropropylene; MMA, methyl methacrylate; PS, polystyrene latex; PVDF, polyvinyledene fluoride.
