# Supplementary material for: Hyperspectral infrared nanoimaging of organic samples based on Fourier transform infrared nanospectroscopy
Source: Nat Commun. 2017 Feb 15;8:14402. doi: 10.1038/ncomms14402 (PMC5316859; doi:10.1038/ncomms14402)
Supplement: Supplementary Information — Supplementary Figures, Supplementary Notes and Supplementary References. [file ncomms14402-s1.pdf]

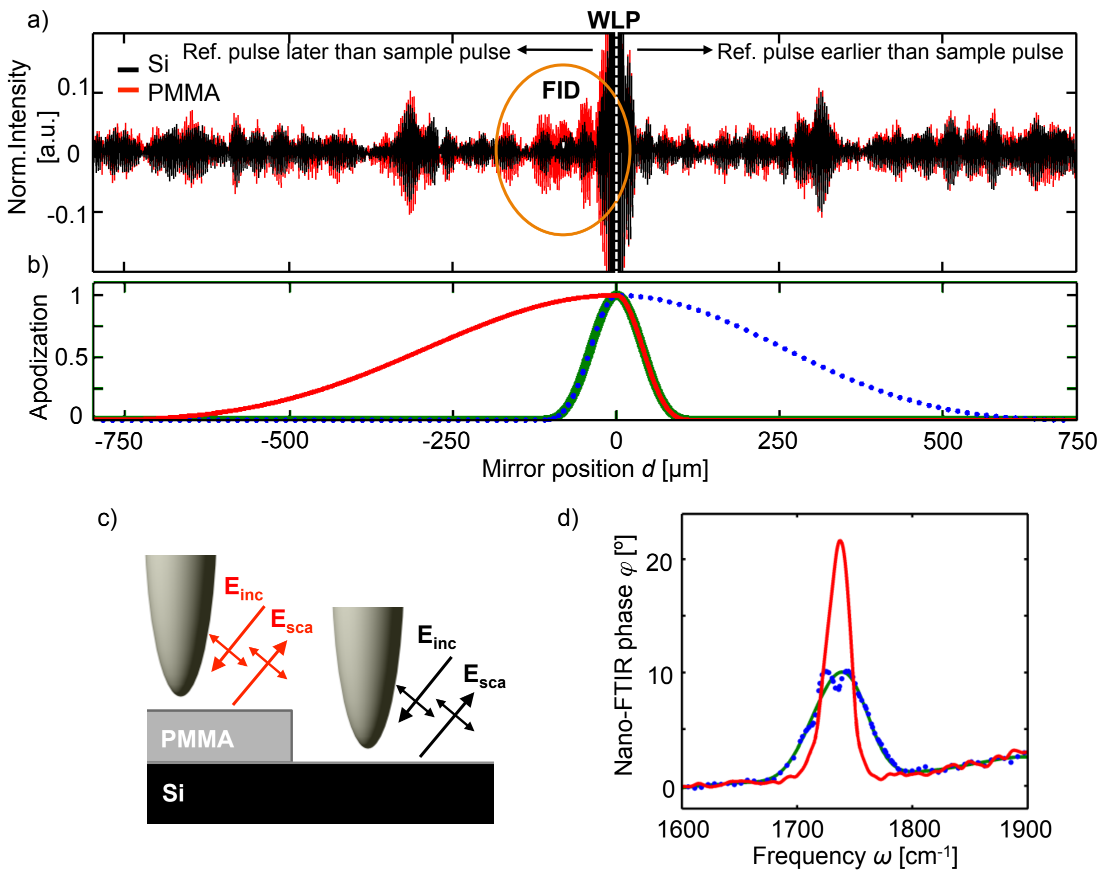

Supplementary Figure 1. Asymmetric interferogram analysis. (**a**) Interferograms of a Poly(methyl methacrylate) (PMMA) film and silicon substrate normalized to the interferogram values at the white light position (WLP). (**b**) Different apodization functions yielding the spectra shown in Supplementary Figure 1d. (**c**) Sketch of the experiment yielding the interferograms shown in Supplementary Figure 1a. (**d**) Phase spectra obtained from interferograms shown in Supplementary Figure 1a by using the apodization functions shown in Supplementary Figure 1b.

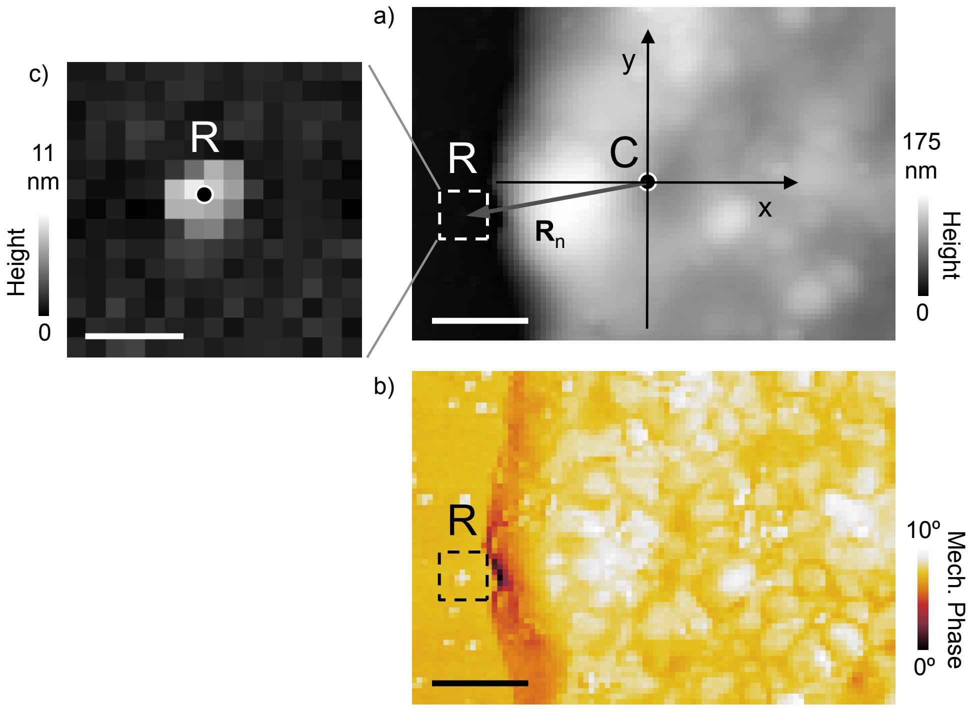

Supplementary Figure 2. Sample drift correction. (**a**) Topography image and (**b**) mechanical atomic force microscopy (AFM) phase image of the area of interest in the polymer blend sample shown in Figure 3. Scale bars, 300 nm. (**c**) Zoom in image of the reference point. Scale bar, 50 nm.

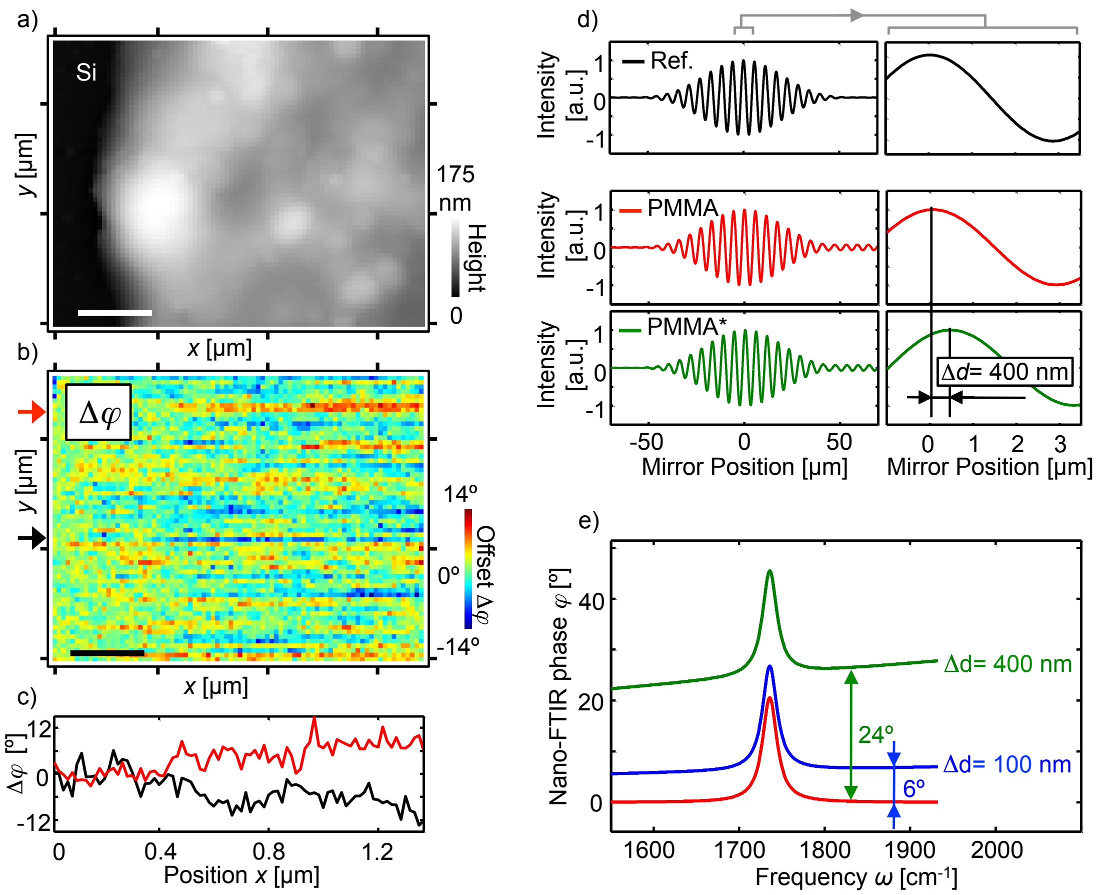

Supplementary Figure 3. Origin of offset fluctuations of phase spectra. (**a**) Topography of the sample area where the hyperspectral data cube was recorded. Scale bar, 300 nm. (**b**) 82 x 62 pixel map showing the offset $\Delta\varphi(x,y)$ between the phase spectra $\varphi^{II}(x,y)$ and φ^{III}(x,y). Scale bar, 300 nm. (**c**) Phase offset $\Delta\varphi$ along the lines marked by the red and black arrows in Supplementary Figure 3b. (**d**) Simulated interferograms for a silicon reference sample (black) and Poly(methyl methacrylate) (PMMA) sample (red, green). The green interferogram is identical to the red interferogram, but shifted by 400 nm in order to mimic a shift of the white light position (WLP), i.e. a drift of the interferometer path length against each other. Right: Zoom into the interferogram around the WLP. (**e**) Calculated phase spectra of PMMA (red and green) normalized to a reference spectrum. Sample and reference spectra were correspondingly obtained by Fourier transform of the interferograms shown in Supplementary Figure 3d. The blue spectrum was obtained by Fourier transform of an interferogram that was shifted by 100 nm compared to the reference interferogram and subsequent normalization.

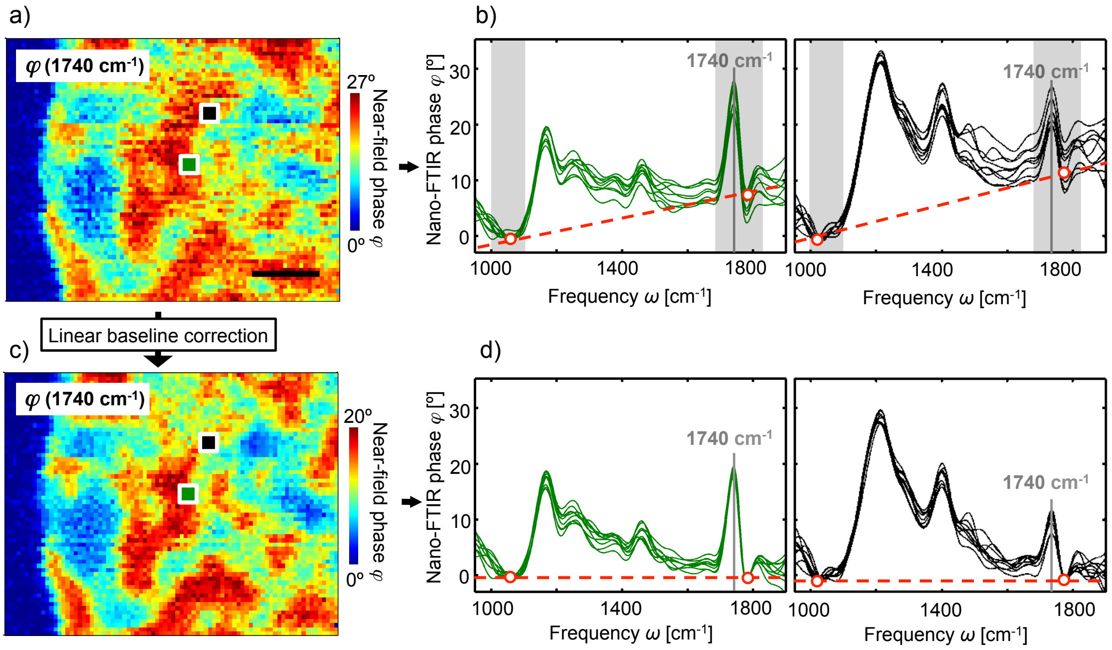

Supplementary Figure 4. Baseline correction. (**a**) Phase image at 1740 cm^{-1} extracted from the hyperspectral data cube shown in Figure 1 prior to linear baseline correction. Scale bar, 300nm. (**b**) nine phase spectra (3x3 pixels) extracted from regions marked by squares in Supplementary Figure 4a. The spectra of the left diagram (green) were taken on a AC (acrylic copolymer)-rich region (marked by green symbol in (a)). The spectra in the right diagram (black) were taken on a FP (fluorine copolymer)-rich region (marked by black symbol in (a)). (**c**,**d**) Same data as in (a) and (b), respectively, but extracted from the hyperspectral data cube after linear baseline correction. The grey areas in (b) show the spectral regions at which the frequencies with minimum phase value where selected. Red dashed lines show the baseline of one of the phase spectra.

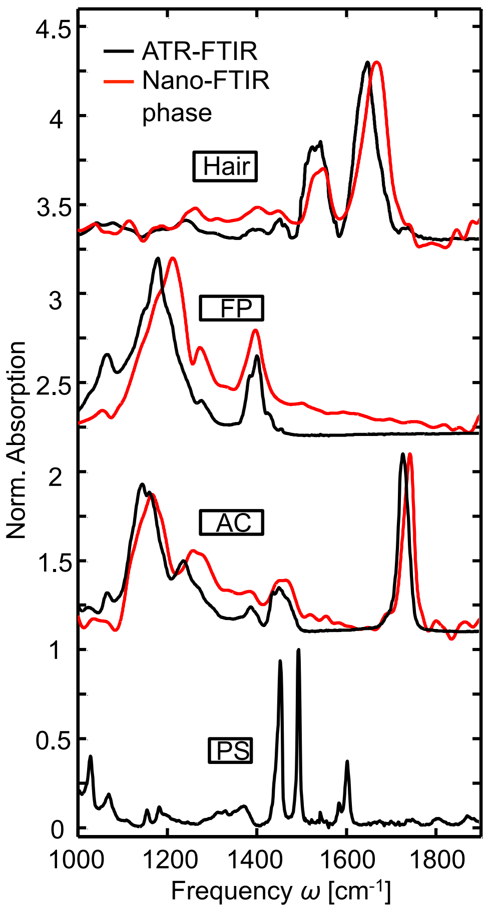

Supplementary Figure 5. Comparison of near-field and far-field infrared spectra. Nanoscale Fourier transform (nano-FTIR) spectra (red, 16 cm^{-1} spectral resolution) and attenuated total reflectance Fourier transform infrared (ATR-FTIR) spectra (black, 4 cm^{-1} spectral resolution) of hair cross-section, fluorine copolymer (FP), acrylic copolymer (AC) and polystyrene latex (PS). For better visibility, we show the spectra with different offsets. The nano-FTIR spectrum of the hair cross-section was recorded on a keratin rich area (see Figure 4 and related discussion), while the ATR-FTIR spectrum was recorded over the whole hair cross-section (including small areas of the embedding epoxy resin, as the ATR crystal was larger than the hair cross section). All ATR-FTIR spectra were recorded after the near-field measurements. We did not observe peaks well above the noise level and thus do not show the nano-FTIR spectra of PS. It seems that the oscillator strength of the PS bonds is much weaker than in other polymers, and thus is not detected with the current nano-FTIR setup.

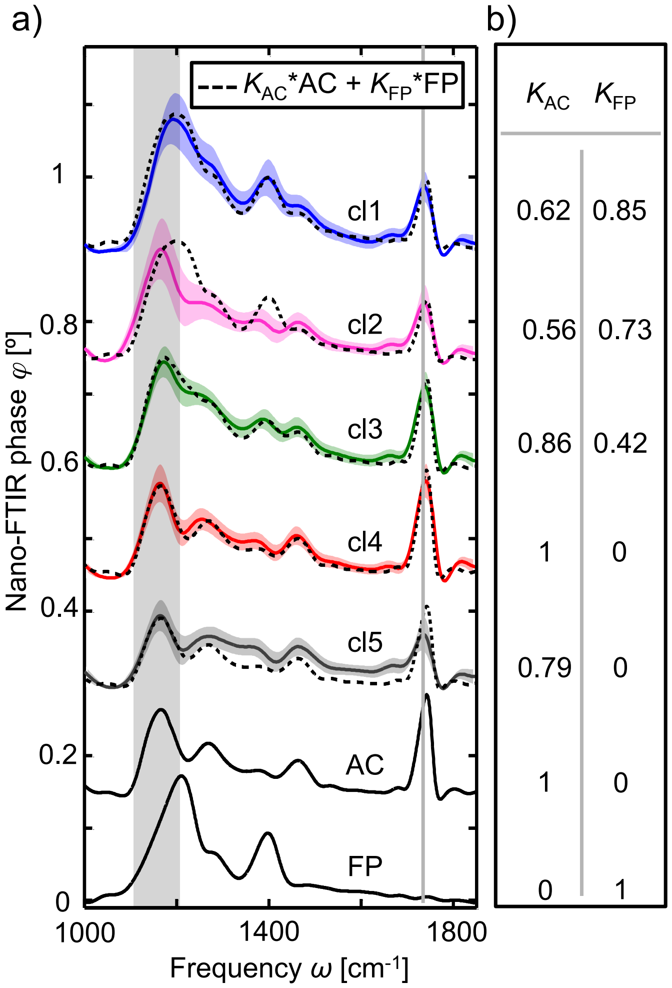

Supplementary Figure 6. Reproducing cluster average nanoscale Fourier transform (nano-FTIR) spectra of Figure 3. (a) The reference nano-FTIR spectra (bottom solid black) of acrylic copolymer (AC) and fluorine copolymer (FP) were linearly superimposed in order to reproduce the average nano-FTIR spectra (colored thin and thick lines, respectively),of the clusters identified in the cluster analysis shown in Figure 3. For each cluster, a best fitting spectrum (dashed black curves) was obtained by multiplication of the reference spectra by factors K_{AC} and K_{FP}, and subsequent addition. The factors K_{AC} and K_{FP} were manually adjusted in order to fit both the height of the peak at 1735 cm^{-1} (exclusive contribution of AC, marked by vertical grey line) and the height of the peak in the range of 1100 - 1200 cm^{-1} (contribution of both AC and FP, marked by the grey area). The applied factors are shown in Supplementary Figure 6b. (**b**) Fitting factors K_{AC} and K_{FP}.

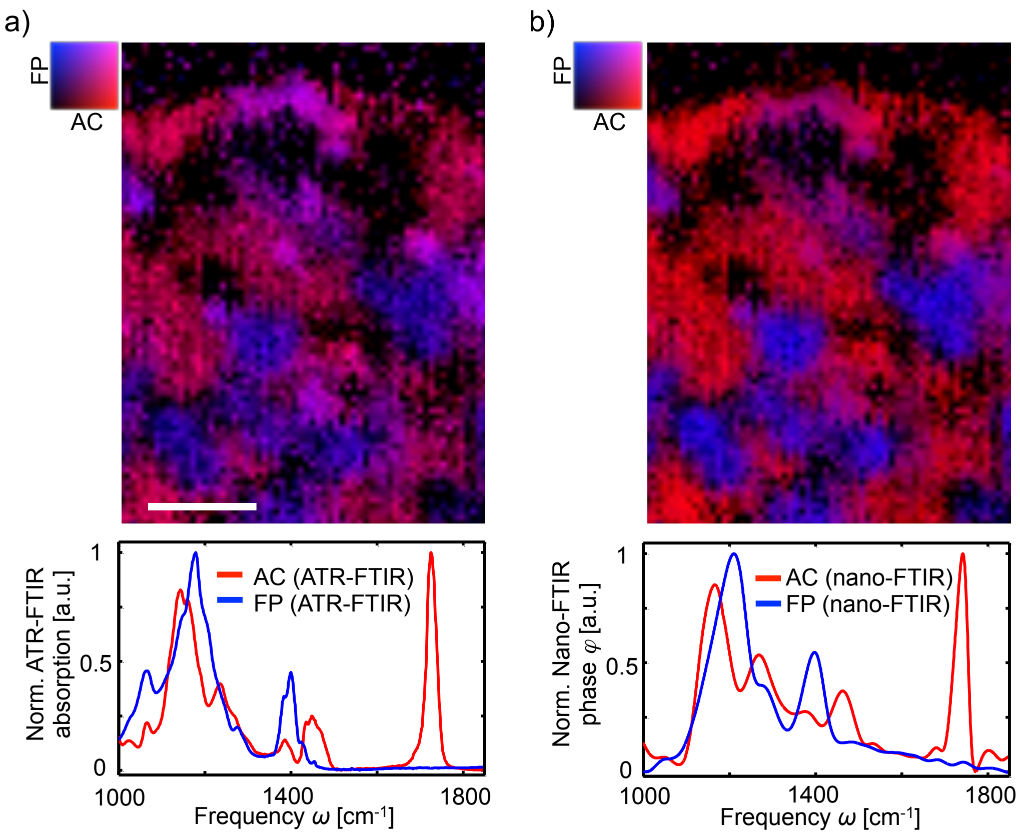

Supplementary Figure 7. Compositional maps obtained with different reference spectra. (**a**) Compositional map (top) of the polymer blend of Figure 3, which was obtained with attenuated total reflectance Fourier transform infrared (ATR-FTIR) reference spectra (bottom, 4 cm^{-1} spectral resolution) of acrylic copolymer (AC, red line) and fluorine copolymer (FP, blue line). Scale bar, 400 nm. (**b**) Compositional map (top) of the polymer blend of Fig. 3, which was obtained with nanoscale Fourier transform infrared (nano-FTIR) reference spectra (bottom, 35 cm^{-1} spectral resolution). All spectra are normalized to their maximum values.

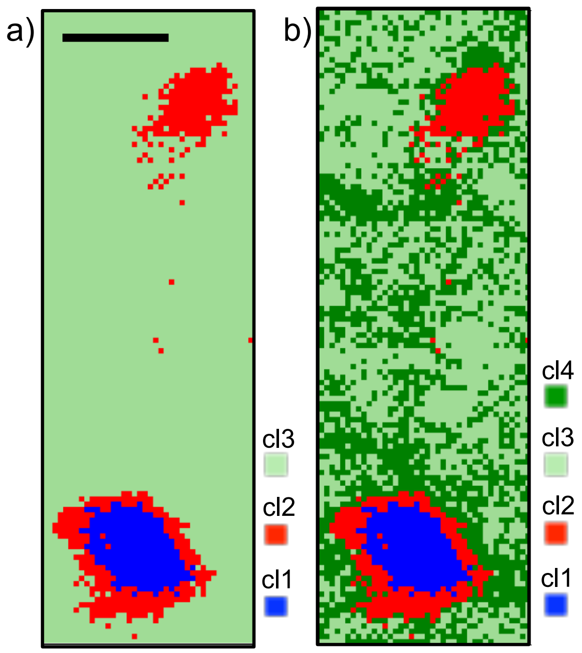

Supplementary Figure 8. Comparison of cluster maps of the hair cross-section. (a) Segmentation with 3 clusters (the map is identical to that in Figure 4b). Scale bar, 300 nm. (b) Segmentation with 4 clusters. The fourth cluster does not reveal any well-defined features. It rather yields a random segmentation of the keratin cluster due to noise-induced spectral variations. Segmentation of the data in more than 3 clusters thus does not provide any additional useful information.

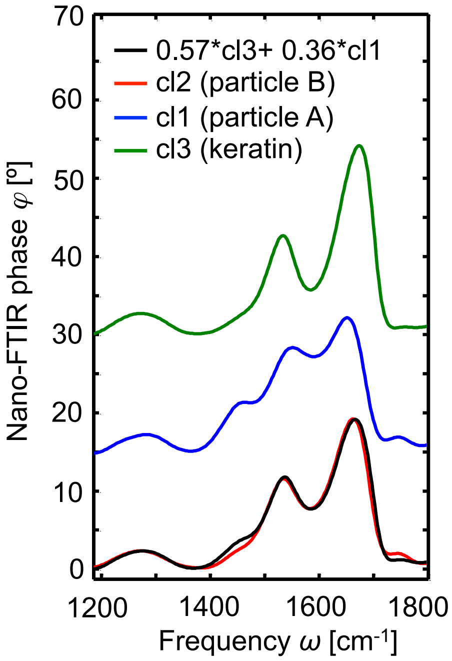

Supplementary Figure 9. Analysis of spectrum B of Figure 4. The nanoscale Fourier transform (nano-FTIR) spectrum of particle B (cluster cl2, red spectrum) can be reproduced by a linear superposition of the nano-FTIR spectrum of melanin (particle A, cluster cl1, blue spectrum) and keratin (cluster cl3, green spectrum). To that end, the nano-FTIR spectra of particle A and keratin were multiplied by the factors $K_{\mathrm{particleA}}$ = 0.36 and K_{keratin} = 0.57, respectively, and then added. The resulting spectrum is shown by the black curve. It matches well the nano-FTIR spectrum (red) of particle B. We conclude that at position B the near field of the tip probes both keratin and melanin. For better visibility, we show the spectra (cluster averages) with different offsets.

Supplementary Note 1. Basic nano-FTIR spectroscopy setup

Our commercial nano-FTIR setup (Neaspec) is based on an atomic force microscope (AFM). It is operated in dynamic mode, where a standard Au-coated tip (PPP-NCSTAu, Nanosensors) is vertically vibrating at the mechanical resonance frequency Ω of the cantilever, in this work at about $\Omega \sim 135$ kHz.

For broadband infrared nanospectroscopy, the AFM tip is illuminated with a coherent mid-infrared beam of an average output power of about 600 μW. The broadband mid-infrared laser beam is generated by a difference frequency generator (Lasnix, Germany) where two near-infrared, 100-fs pulse trains from a fibre-laser system (FemtoFiber pro IR and SCIR, Toptica Germany) are superimposed in a GaSe crystal. This mid-infrared source emits a continuous spectrum with a usable width up to 350 cm^{-1}, which can be tuned within the limits 1000–1900 cm^{-1} dependent on difference frequency generator settings (for example, the crystal orientation).

The light backscattered from the tip is recorded with an asymmetric Fourier transform infrared (FTIR) spectrometer based on a Michelson interferometer, while unavoidable background signals are efficiently suppressed by demodulating the detector signal at a higher harmonic of the tapping frequency, $n\Omega$ [1]. FTIR spectroscopy of the tip-scattered light is performed by recording the demodulated detector signal I as a function of the reference mirror position d. In all nano-FTIR spectra presented in this work, the demodulation order was $n = 3$. For simplicity, the index n is omitted in equations. Because of the asymmetric spectrometer (that is, the sample is located in one interferometer arm), Fourier transformation of the interferogram $I(d)$ subsequently yields the near-field amplitude $s_{\mathrm{s}}(\omega)$ and phase $\varphi_{\mathrm{s}}(\omega)$ spectra, that is the complex valued near-field spectrum $E_{\mathrm{s}}(\omega) = s_{\mathrm{s}}(\omega)e^{i\varphi_{\mathrm{s}}(\omega)}$. Note that zero filling of the interferogram and apodization are applied prior to FT, analogue to far-field FTIR spectroscopy [2].

For normalizing the near field spectrum, the tip is typically positioned on a clean area of the substrate (Si or Au) and a reference interferogram is recorded. Subsequent FT yields the near-field reference $E_{\mathrm{ref}}(\omega) = s_{\mathrm{ref}}(\omega)e^{i\varphi_{\mathrm{ref}}(\omega)}$, that is, the near-field spectrum of the clean substrate. By complex-valued division, the normalized near field spectrum is obtained $E(\omega) = E_{\mathrm{s}}(\omega)/E_{\mathrm{ref}}(\omega)$, i.e. normalized amplitude $s_{\mathrm{s}}(\omega)/s_{\mathrm{ref}}(\omega)$ and phase $\varphi_{\mathrm{s}}(\omega) - \varphi_{\mathrm{ref}}(\omega)$ spectra.

Supplementary Note 2. Increase of data acquisition speed – asymmetric interferogram analysis

In far-field FTIR spectroscopy a symmetric Michelson interferometer is used, which yields interferograms that are symmetric in respect to the white light position (WLP, corresponding to the position of the reference mirror at which the optical path lengths of both interferometer arms are equal). In contrast, nano-FTIR spectroscopy uses an asymmetric Michelson interferometer, where the sample (together with the tip) is located in one of the interferometer arms. For causality reasons, the interferograms obtained from the sample are asymmetric [3,4] and only one half of the interferogram contains information about the sample. We demonstrate this aspect in Supplementary Figure 1. To this end, we recorded interferograms of a thin Poly(methyl methacrylate) (PMMA) film on silicon (red line in Supplementary Figure 1a) and of the clean silicon substrate next to it (black line in Supplementary Figure 1a). The experiment is sketched in Supplementary Figure 1c. We observe that both interferograms show strong signal oscillations even far away from the WLP. They can be explained by the spectrally sharp

mid-infrared absorption of vapor water. Due to the presence of the water vapor in both interferometer arms, the signal oscillations are symmetric to the WLP. Further, they are the same for both the PMMA and the reference sample. Both findings confirm that they do not contain specific information about the PMMA sample. On the other hand, on the left side of the WLP we observe significant differences (marked by orange circle in Supplementary Figure 1a) between the PMMA and the reference (Si) interferograms. They are caused by the free-induction decay (FID) of the molecular vibrations in the PMMA sample [3].

By FT of the two interferograms shown in Supplementary Figure 1a and subsequent normalization of the PMMA spectrum to the Si spectrum, we obtain the nano-FTIR phase spectrum of PMMA. Due to this normalization procedure (see S1.1 for details) the water absorption lines are efficiently removed from the PMMA spectrum. However, we cannot exclude that spatial and temporal fluctuations of the vapor water in the interferometer arms increase the noise in the nano-FTIR spectra.

In order to analyze the information encoded at the two sides of the WLP, we applied different asymmetric apodization windows (three-term Blackman-Harris [2]) prior to FT, which are shown in Supplementary Figure 1b (solid red and dotted blue curves). For comparison, we also applied a narrow symmetric apodization window (green solid curve in Supplementary Figure 1b). The obtained nano-FTIR spectra are shown in Supplementary Figure 1d

The green nano-FTIR spectrum clearly reveals the absorption peak of the C=O vibration at 1740 cm^{-1} [5]. The peak is rather broad due to the low spectral resolution owing to the narrow apodization window. The same spectrum (blue dotted curve in Supplementary Figure 1d) is obtained when the width of the apodization function is increased on the right side of the WLP (blue dotted graph in Supplementary Figure 1b). In other words, the spectral resolution is not improved, which can be explained by the absence of a FID at the right side of the interferogram of PMMA. However, increasing the width of the apodization function on the left side of the WLP (red function in Supplementary Figure 1b) yields an improved spectral resolution, as can be seen by narrowing of the absorption peak in the red spectrum compared to the blue dotted spectrum (Supplementary Figure 1d). The increasing spectral resolution also comes along with an increase of the height of the red peak compared to the green peak, which is an intrinsic feature of Fourier transform spectroscopy [6].

Our analysis clearly shows that recording of the right part of the interferogram (typically done in standard FTIR) does not provide any spectral information and thus can be skipped in nano-FTIR spectroscopy.

We also point out that the spectral resolution of the asymmetric nano-FTIR spectra is determined by the length of the interferogram on the left side of the WLP, i.e. the side of the interferogram that contains the FID.

Supplementary Note 3. Sample drift correction

In the nano-FTIR setup used in this work the AFM tip is fixed (it only oscillates vertically), while the sample is scanned. To this end, the sample is placed on a linearized scanner with 100 by 100 μm lateral scan range and capacitive position sensors. The methodology for the sample drift correction is described in the following.

First, a topography image of the area of interest is taken (see Supplementary Figure 2a) and the centre of the hyperspectral image to be recorded is defined and marked as $\mathbf{C}_0 = (0{,}0)$ in the scanner software. Further, with the help of the mechanical phase image (see Supplementary Figure 2b) a small topographical feature (about 40 nm in lateral diameter and 10 nm in height) inside the area of interest is selected as a reference point, R. A small topography image of 150 by 150 nm is recorded in about 5 s (see Supplementary Figure 2c) and the position of the selected reference point in the coordinate system of the scanner is measured, $\mathbf{R}_0$. Then, the first few lines of the data cube are recorded. After a time of about 4-5 minutes we interrupt the data acquisition and record again the small topography image and the position of the reference point is measured, $\mathbf{R}_1$. The difference between the old and new position of the reference point yields the sample drift that occurred during the line scans, $\mathbf{D}_1 = \mathbf{R}_1 - \mathbf{R}_0$. Accordingly, we update in the scanner software the centre coordinates of the image, $\mathbf{C}_1 = \mathbf{C}_0 + \mathbf{D}_1$, and continue with the data cube acquisition. This procedure is repeated until the whole data cube is recorded; at the n-th repetition we measure the sample drift, $\mathbf{D}_\mathrm{n} = \mathbf{R}_\mathrm{n} - \mathbf{R}_{\mathrm{n}-1}$, and update the centre coordinates to $\mathbf{C}_\mathrm{n} = \mathbf{C}_{\mathrm{n}-1} + \mathbf{D}_\mathrm{n}$.

The new position of the tip can be set by the scanner software in discreet steps of 10 nm, that is, with an error of about 5 nm. The repeatability of the physical positioning is of about 2 nm, which is determined by the capacitive sensor of the commercial scanner. Thus the maximum error of the re-positioning is of about 7 nm. In all recorded data cubes, the average sample drift $|\mathbf{D}_{\mathrm{AVG}}|$ was less than 27 nm per 4 minutes, which is smaller than the spatial resolution of about 30 nm [5,7]. For that reason, our method allows for the recording of nearly drift-free data cubes. Among others, this achievement is essential for reliable spectral stitching of different data cubes of one and the same area.

For the image shown in Supplementary Figure 2a, the repositioning procedure was performed every 2 lines (i.e. every 4-5 minutes), that is, 30 times during the recording of the complete image of 82x62 pixels. The recording of the small topography image plus the calculation and repositioning to the updated centre coordinates took about 20 seconds, which yields an accumulated time of about 10 minutes.

Supplementary Note 4. Normalization of nano-FTIR spectra in Figure 4

In case of the hair cross section shown in Figure 4, the recorded hyperspectral data cube did not contain reference spectra due to the lack of an appropriate reference surface. For that reason, a small piece (about 2 mm in diameter) of a clean silicon wafer was placed about 300 μm next to the hair cross-section in order to record reference spectra.

As before with the polymer sample, we performed sample drift correction every 4 lines with the help of a reference feature R on the hair sample. After 20 lines, yielding the partial data cube $\varphi_\mathrm{s}^{\mathrm{k},1}(x, y, \omega)$, we stopped the data acquisition at the sample position (x_1,y_1) relative to R, in order to record a reference spectrum (note that the recording of 20 lines (40 pixels/line) required a time of about 22 minutes, in which the DFG output spectrum did not exhibit noticeable fluctuations). To that end, we moved the center of the silicon surface below the tip (about 10 seconds) and recorded an about 200 x 200 nm size topography image, in order to assure that the surface was clean (about 20 seconds). Subsequently, we recorded the reference phase spectrum $\varphi_{\mathrm{ref}}^{\mathrm{k},1}(\omega)$ (average over several individual reference spectra with a total acquisition time of 30 seconds). We then moved the sample position (x_1,y_1) below the tip, which took about 3 minutes.

To that end, we first moved the hair cross section roughly below the tip by using the optical image of sample and cantilever, which was obtained with the optical microscope that is integrated in the NeaSNOM. By recording a topography image of the hair sample, we identified the reference feature R, which allows for moving the sample position (x_1,y_1) exactly below the tip. We then continued the data cube acquisition for another 20 lines, followed by an interruption for recording the next reference spectrum, yielding $\varphi_{\mathrm{s}}^{\mathrm{k},2}(x,y,\omega)$ and $\varphi_{\mathrm{ref}}^{\mathrm{k},2}(\omega)$, respectively. This procedure was repeated 5 times until the complete data cube was recorded. The nano-FTIR spectra of each partial data cube were normalized to the corresponding reference spectrum according to $\varphi^{\mathrm{k,j}}(x,y,\omega) = \varphi_{\mathrm{s}}^{\mathrm{k,j}}(x,y,\omega) - \varphi_{\mathrm{ref}}^{\mathrm{k,j}}(\omega)$. The recording of each reference spectrum, including the related sample repositioning, process took about 4 minutes, which resulted in an accumulated extra time of about 20 minutes for the whole data cube.

Supplementary Note 5. Origin of offset fluctuations of phase spectra

To clarify the origin of the fluctuations of the offset $\Delta\varphi$ between the phase spectra of adjacent data cubes (as observed in Figure 2), we plot in Supplementary Figure 3b the offset $\Delta\varphi$ between the phase spectra φ^{II} and φ^{III} comprising the hyperspectral data cube of Figure 1d as a function of spatial position (x,y), yielding a phase offset map $\Delta\varphi(x,y)$. For interpreting the data, note that the individual phase spectra of each horizontal line were normalized to the averaged reference spectrum of the same line, which was obtained on the silicon area (black area on the left side of the topography image shown in Supplementary Figure 3a). For each line, we observe that $\Delta\varphi$ slightly fluctuates around 0 degree on the silicon area, which can be attributed to fluctuations of the effective interferometer path length caused by air turbulences or convection. For increasing distance from the silicon area, we find that $\Delta\varphi$ in numerous lines tends to either increase or decrease (as can be seen more clearly for the two line profiles shown in Supplementary Figure 3c). This observation lets us assume that $\Delta\varphi$ originates from a drift of the WLP caused by a relative drift of the interferometer path lengths.

To elucidate how the drift of the WLP shifts the offset of the normalized phase spectrum, we calculated theoretical reference and PMMA interferograms (black and red curves in Supplementary Figure 3d). The reference interferogram was calculated by Fourier transformation of a bandwidth-limited virtual laser output spectrum. The PMMA interferogram was calculated by Fourier transformation of a complex-valued infrared spectrum of a Lorentz oscillator at 1730 $\mathrm{cm^{-1}}$ (being a proxy for the C=O stretching) illuminated by the virtual laser output spectrum. To mimic a shift of the WLP, i.e. a drift of the interferometer path length against each other, we shift the sample interferogram by 400 nm to the right, yielding the green interferogram in Supplementary Figure 3d. Fourier transform of the shifted (green) interferogram and normalization to the Fourier transform of the reference (black) interferogram of Supplementary Figure 3d yields a phase spectrum (green curve in Supplementary Figure 3e), which baseline is tilted around $\omega = 0$ $\mathrm{cm^{-1}}$ leading to a phase offset $\Delta\varphi$ of about 24 degrees at around 1730 $\mathrm{cm^{-1}}$ compared to the original phase spectrum (red curve in Supplementary Figure 3e). We also show in Supplementary Figure 3e a phase spectrum (blue) obtained by Fourier transform of an interferogram that was shifted by 100 nm compared to the reference interferogram (black curve in Supplementary Figure 3d). The smaller interferometer shift results in a smaller phase offset $\Delta\varphi$ of about 6 degree, while the tilting of the baseline is barely recognized. The calculations of Supplementary

Figure 3d,e support our explanation that the offset of normalized phase spectra is caused by an interferometer drift between the recording of the reference and sample spectra. A WLP drift as small as 100 nm yields a phase offset of about 6 degrees, which is in the range of the experimentally observed phase offsets (Supplementary Figure 3b). Taking into account that the time between reference and sample measurement in one line of Supplementary Figure 3b can take up to 136 s (one line consists of 82 pixel, while one spectrum requires 1.66 s), the WLP was drifting by about 50 nm per minute. Such drift is relatively small and unavoidable, considering that the interferometer was not stabilized and exposed to the lab environment without any enclosure. A drift of 50 nm relative to an interferometer path length of about 6 cm corresponds to a relative drift of about 10^{-6}. Importantly, according to our calculations, for small phase offsets similar to the ones observed in our experiments (i.e. about 6 degrees) the tilting of the baseline is barely noticeable. Thus, as shown in Figure 2, we correct the phase offsets by shifting the complete phase spectra.

Supplementary Note 6. Baseline correction

Baseline correction is a basic correction method in far-field FTIR, where different algorithms are used (i.e. linear, polyline, etc.) to define the baseline to be corrected [6]. We apply a linear baseline correction to correct each individual broadband phase spectrum. The advantage of baseline correction is demonstrated in Supplementary Figure 4, where we compare phase images and phase spectra (extracted from hyperspectral data cube of Figure 1d) before and after baseline correction.

The baseline correction is done as follows. Based on far-field FTIR spectra of the sample components, two spectral regions are selected at which all the components have at least one non-absorbing (or negligibly absorbing) frequency. For the data cube recorded at the polymer film (shown in Figure 1 and Supplementary Figure 4) we selected the spectral regions 1000 - 1100 cm^{-1} and 1700 - 1850 cm^{-1} (marked as grey areas in Supplementary Figure 4b), where the ATR-FTIR spectra of all three polymer components exhibit frequencies with negligibly small absorption (see Supplementary Figure 5). At each pixel of the hyperspectral data cube, the frequencies are determined where the phase (corresponding to absorption) in the two selected spectral regions assumes its minimum value. An example of this procedure is illustrated in Supplementary Figure 4b, where the two frequencies used for baseline correction are marked by red open circles. Note that the evaluated spectral regions are the same for all pixels, while the selected frequencies may be different. The line determined by the two selected frequencies is defined as the baseline (marked as dashed red line in Supplementary Figure 4b) and is subtracted from the phase spectrum (illustrated in Supplementary Figure 4d). The baseline correction is performed individually for all phase spectra of the hyperspectral data cube. It allows for reliable and automatic baseline correction of the individual spectra, provided that all spectra have at least one non-absorbing frequency in the selected spectral ranges. Supplementary Figure 4d clearly shows that the linear baseline correction does not alter the spectral features of individual spectra. However, most importantly, it reduces fluctuations between neighboring spectra (compare Supplementary Figures 4b and 4d), and hence significantly improves the SNR of the images extracted from the hyperspectral data cube (compare Supplementary Figures 4a and 4c).

Supplementary Note 7. Compositional map of the polymer blend using ATR-FTIR references

We take the hyperspectral data cube of the polymer blend (shown in Figure 1d) and apply the inter-spectral distance imaging method (same as applied in Figure 3) using external reference spectra of AC and FP polymers obtained by ATR-FTIR spectroscopy. The ATR-FTIR reference spectra and the obtained compositional map are shown in Supplementary Figure 7a. For comparison, external nano-FTIR reference spectra and the obtained compositional map are shown in Supplementary Figure 7b (same data as in Figure 3d).

The nano-FTIR reference spectra have the same spectral resolution as the nano-FTIR spectra of the hyperspectral data cube (i.e. 35 cm^{-1}, see Supplementary Figure 5 for higher resolution AC and FP nano-FTIR spectra). In order to test the general approach of using standard far-field FTIR reference spectra (i.e. taken from a standard FTIR database), we used ATR-FTIR reference spectra with a spectral resolution of 4 cm^{-1} typically used in standard far-field FTIR spectroscopy.

Most important, we observe that the compositional maps shown in Supplementary Figures 7a and 7b are very similar and reveal the same blue, red, black and purple areas, demonstrating that standard reference spectra can be applied for automatic compositional mapping with the use of hyperspectral data cubes based on nano-FTIR spectroscopy.

The SNR and the color contrast in Supplementary Figure 7a is a bit lower than in Supplementary Figure 7b, which can be explained by the slight spectral shifts between ATR-FTIR and nano-FTIR spectra [7–9]. Note that original ATR-FTIR spectra (normalized to their maximum absorbance but without any spectral shifting or other post-processing) were used for the analysis.

Supplementary References

1. Ocelic, N., Huber, A. & Hillenbrand, R. Pseudoheterodyne detection for background-free near-field spectroscopy. *Appl. Phys. Lett.* **89,** 101124-1-101124–3 (2006).

2. Herres, W. & Gronholz, J. Understanding FT-IR data processing. *Part* **1,** 352–356 (1984).

3. Amarie, S., Ganz, T. & Keilmann, F. Mid-infrared near-field spectroscopy. *Opt. Express* **17,** 21794–21801 (2009).

4. Xu, X. G. & Raschke, M. B. Near-field Infrared Vibrational Dynamics and Tip-Enhanced Decoherence. *Nano Lett.* **13,** 1588–1595 (2013).

5. Huth, F. *et al.* Nano-FTIR Absorption Spectroscopy of Molecular Fingerprints at 20 nm Spatial Resolution. *Nano Lett.* **12,** 3973–3978 (2012).

6. Griffiths, P. R. & de Haseth, J. A. *Fourier Transform Infrared Spectrometry. Chemical Analysis: A Series of Monographs on Analytical Chemistr and Its Applications* (Wiley, 2007).

7. Amenabar, I. *et al.* Structural analysis and mapping of individual protein complexes by infrared nanospectroscopy. *Nat. Commun.* **4,** 2890 (2013).

8. Govyadinov, A. A., Amenabar, I., Huth, F., Carney, P. S. & Hillenbrand, R. Quantitative Measurement of Local Infrared Absorption and Dielectric Function with Tip-Enhanced Near-Field Microscopy. *J. Phys. Chem. Lett.* **4,** 1526–1531 (2013).

9. Mastel, S., Govyadinov, A. A., de Oliveira, T. V. A. G., Amenabar, I. & Hillenbrand, R. Nanoscale-resolved chemical identification of thin organic films using infrared near-field spectroscopy and standard Fourier transform infrared references. *Appl. Phys. Lett.* **106,** 23113 (2015).
